# AMP-Activated Kinase AMPK Is Expressed in Boar Spermatozoa and Regulates Motility

**DOI:** 10.1371/journal.pone.0038840

**Published:** 2012-06-14

**Authors:** Ana Hurtado de Llera, David Martin-Hidalgo, María C. Gil, Luis J. Garcia-Marin, María J. Bragado

**Affiliations:** Research Group of Intracellular Signalling and Technology of Reproduction (SINTREP), Veterinary School, University of Extremadura, Caceres, Spain; Boston University Medical Center, United States of America

## Abstract

The main functions of spermatozoa required for fertilization are dependent on the energy status and metabolism. AMP-activated kinase, AMPK, acts a sensor and regulator of cell metabolism. As AMPK studies have been focused on somatic cells, our aim was to investigate the expression of AMPK protein in spermatozoa and its possible role in regulating motility. Spermatozoa from boar ejaculates were isolated and incubated under different conditions (38,5°C or 17°C, basal medium TBM or medium with Ca^2+^ and bicarbonate TCM, time from 1–24 hours) in presence or absence of AMPK inhibitor, compound C (CC, 30 µM). Western blotting reveals that AMPK is expressed in boar spermatozoa at relatively higher levels than in somatic cells. AMPK phosphorylation (activation) in spermatozoa is temperature-dependent, as it is undetectable at semen preservation temperature (17°C) and increases at 38,5°C in a time-dependent manner. AMPK phosphorylation is independent of the presence of Ca^2+^ and/or bicarbonate in the medium. We confirm that CC effectively blocks AMPK phosphorylation in boar spermatozoa. Analysis of spermatozoa motility by CASA shows that CC treatment either in TBM or in TCM causes a significant reduction of any spermatozoa motility parameter in a time-dependent manner. Thus, AMPK inhibition significantly decreases the percentages of motile and rapid spermatozoa, significantly reduces spermatozoa velocities VAP, VCL and affects other motility parameters and coefficients. CC treatment does not cause additional side effects in spermatozoa that might lead to a lower viability even at 24 h incubation. Our results show that AMPK is expressed in spermatozoa at high levels and is phosphorylated under physiological conditions. Moreover, our study suggests that AMPK regulates a relevant function of spermatozoa, motility, which is essential for their ultimate role of fertilization.

## Introduction

The spermatozoon is a germ cell that is highly specialized for cellular processes, motility, capacitation, hyperactivation and acrosome reaction that promote its essential function of oocyte fertilization. All these cellular processes are dependent on the energetic cellular state, determined by the ratio between cellular AMP and ATP, [1], [2] and regulated by biochemical mechanisms such as phosphorylation of proteins. Spermatozoa possess an elaborated intracellular compartmentalization and, in the last phase of development, are transcriptionally inactive and thus unable to synthesize proteins. Therefore, the intracellular pathways that regulate those cellular processes based in post translation modifications of pre-existing proteins, such as phosphorylation, catalyzed by kinases, are especially important in these germ cells.

The AMP activated protein kinase AMPK is an evolutionarily conserved serine/threonine kinase that acts as a sensor that detects the cell energy state and subsequently regulates metabolism [3]. AMPK is a heterotrimeric protein that has a catalytic α and two regulatory subunits, β and γ. One of the essential features of the AMPK kinase as a sensor and metabolic regulator is its extreme sensitivity to AMP, as any increase in the ratio AMP/ATP that means a decrease in cellular energy state, activates AMPK [3], [4]. Optimal allosteric activation of AMPK, which is induced by binding of AMP to the γ subunit, requires formation of the αβγ complex [3], [5], [6], [7]. In addition to allosteric activation by AMP, phosphorylation of the Thr^172^ residue, located at the critical activation loop of the α subunit, is absolutely required for full AMPK activation [8]. Phosphorylation of AMPK is carried out by an upstream kinase that functions as a tumor suppressor called LKB1 (Peutz-Jerhers protein). Additionally, AMP binding to AMPK inhibits dephosphorylation of Thr^172^. When AMPK becomes activated it stimulates catabolic pathways that produce ATP, while simultaneously inhibits ATP-consuming anabolic pathways [9], [10], therefore the overall metabolic consequences of AMPK activation is the maintenance of cellular energy stores. The best known substrates of AMPK are acetyl CoA-carboxylase [11] and hydroxymethylglutaryl CoA-reductase, which are the most regulated enzymes in the synthesis pathways of fatty acids and cholesterol, respectively, and also the phosphofructokinase 2, key enzyme in the carbohydrate metabolism [3], [4], [12]. However, AMPK is a ser/thr kinase and may regulate processes outside metabolism [13]. Recently it has been demonstrated that AMPK activity is also induced by several types of stimuli involving metabolic stresses such as glucose deprivation, hypoxia, ischemia, oxidative or hyperosmotic stress [4], heat shock or alterations of mitochondrial oxidative production [3], [14], [15]. Some AMPK stimuli as hyperosmotic stress do not alter AMP/ATP ratio suggesting that other mechanisms are involved in its activation. Recent studies identified the calcium calmodulin-dependent protein kinase kinase (CaMKK) as an enzyme that also activates AMPK [3], [4] by an increase in calcium concentration, with no appreciable change in the AMP/ATP ratio. As CaMKK is expressed at very high levels in the central nervous system and to a lesser extent in other tissues, it is postulated that the intracellular pathway of AMPK may be regulated by multiple mechanisms that are possibly cell type specific.

All AMPK studies have been conducted exclusively in somatic cells, and there is no work performed in spermatozoa to date. However, there are some studies in germ cells with AMPK-related kinases, as recently it has been reported that a shorter isoform of LKB1, called LKB1s, is expressed predominantly in haploid sperm cells from testes of mammals [16]. LKB1s knockout mice have a dramatic reduction in the number of mature spermatozoa in the epididymis, and the few spermatozoa produced are not mobile, have an abnormal head morphology and resulted sterile [16]. These data suggest that this variant of the LKB1 has a crucial role in spermiogenesis and fertility in mice. Moreover, members of “ser/thr kinase testis specific" TSSK family, which belongs to the AMPK branch in the human kinome tree, have been identified in human spermatozoa: TSSK2, TSKS and SSTK [17]. Deletion of TSSK1 and 2 causes male infertility in chimera mice due to haploinsufficiency [18].

Besides the mentioned upstream kinases LKB1 and CaMKK in somatic cells, it has been demonstrated that protein kinase A (PKA) regulates AMPKα activity by phosphorylation at Ser-173 in mouse adipocytes [19]. The fact that PKA pathway, which is stimulated by HCO_3_^−^ [20] or Ca^2+^ in spermatozoa, highly regulates spermatozoa function [21], further supports the idea that AMPK might play a role in male germ cells.

The study of mechanisms by which spermatozoa regulates their energy status through AMPK is very important for the understanding of the ability of these germ cells to survive and adapt to external extreme conditions such as the transit through the female genital tract. In this regard, our hypothesis is that AMPK would act a sensor molecule of the cell energetic state of spermatozoa and subsequently would regulate their most relevant cellular processes required for successful fertilization. Therefore, the aim of this work is to study the protein expression and activity status of AMPK in spermatozoa, and in addition to investigate its role in the regulation of one of the most important functional procesess of these germinal cells: motility.

## Results

### Expression of AMP Activated Kinase, AMPK, in Boar Spermatozoa

**Figure 1:**
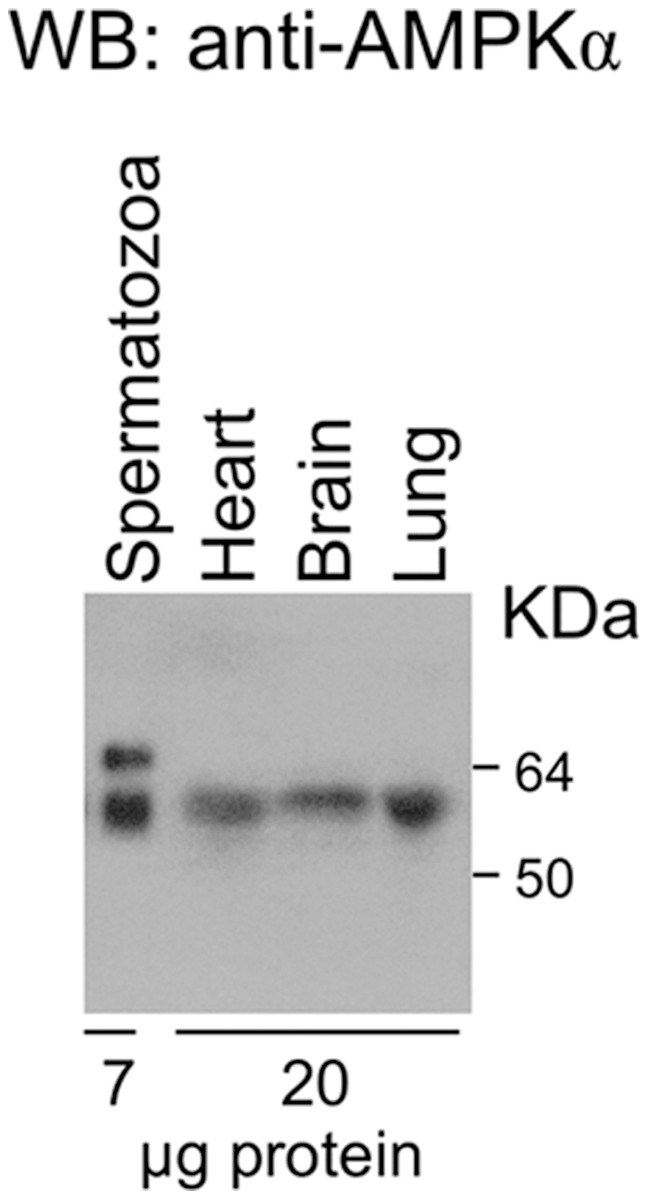
Expression of AMP-activated kinase protein, AMPK, in boar spermatozoa . Protein from boar spermatozoa lysates (lane 1) were analyzed by western blotting using anti-AMPKα as primary antibody. Other porcine tissues were homogenized and used as positive controls (hearth, lane 2; brain, lane 3 and lung, lane 4). Molecular weight markers are indicated in kDa and the amount of protein (µg) loaded into each lane is shown at the bottom. This experiment was performed 7 times and a representative film is shown.

A western blotting analysis was performed to determine whether AMPK is expressed in boar spermatozoa. Two cross reactive bands are detected in boar spermatozoa lysates using a specific antibody against the α catalytic subunit of AMPK (Figure 1, lane 1). Positive controls for this antibody were tested including proteins extracted from other porcine tissues such as brain, lung or heart, where a single reactive band is detected and corresponds to the smaller molecular weight band identified in spermatozoa. Negative control for this antibody was performed omitting the primary antibody and blot was probed with secondary antibody (anti-rabbit-HRP) only. Results show that no band is detected with the secondary antibody and confirm that bands visualized are due to the AMPKα antibody used (data not shown). It is interesting to mention that the expression level of AMPK protein detected in male germ cells (7 µg proteins were loaded into the SDS-PAGE) is likely higher than in somatic cells derived from other porcine tissues (20 µg proteins).

### AMPK is Phosphorylated at Thr^172^ (Activated) in Basal Physiological Conditions in Boar Spermatozoa

**Figure 2:**
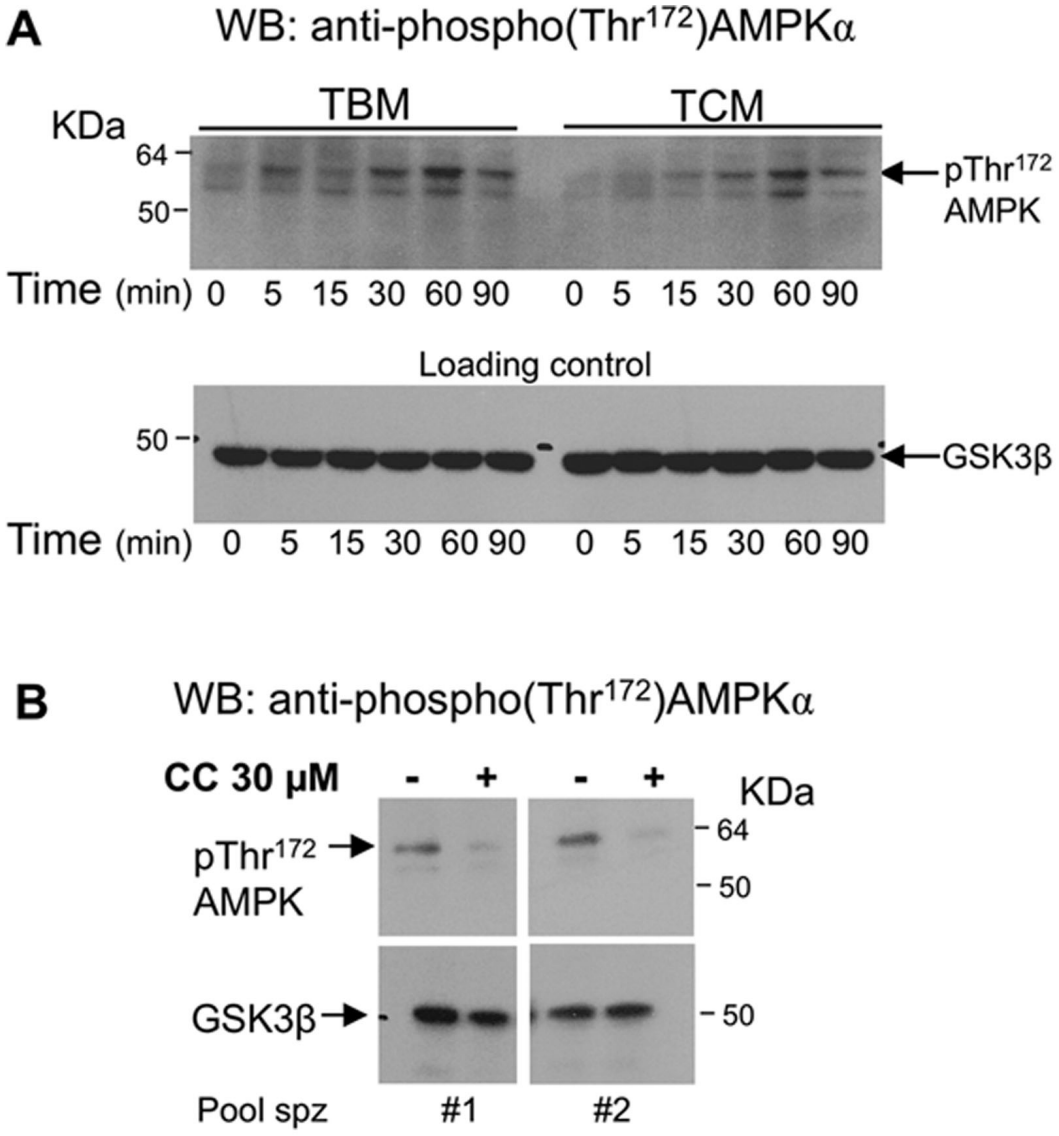
Phosphorylation of AMPK at Thr^172^ is regulated at physiological temperature in boar spermatozoa and is effectively inhibited by the compound C. 2A: Spermatozoa were incubated in TBM or TCM medium at 38.5°C for indicated times and then lysed. Samples at 17°C were considered as time 0. Proteins (20 µg) from lysates were analyzed by western blotting using anti-phospho-Thr^172^-AMPKα as primary antibody. Arrow indicates the cross-reactive band of phospho-Thr^172^AMPK, recognized by the anti-AMPKα. This experiment was performed 6 times and a representative film is shown. 2B: AMPK phosphorylation was evaluated in spermatozoa incubated in TBM in the presence (+) or absence (−) of AMPK inhibitor, compound C (CC 30 µM) at 38.5°C during 24 h. This experiment was performed 3 times and a representative film is shown. Loading controls were performed for each experiment in the same membranes (with different time of exposure) using anti-GSK3β antibody and are showed at lower panels in 2A and 2B.

The level of phosphorylation of AMPK in Thr^172^ was analyzed at physiological temperature of boar spermatozoa (38.5°C) as an assessment of its enzymatic activity. As seen in Figure 2A, two cross-reactive bands are detected with anti-phospho-Thr^172^-AMPK antibody, being the upper band the AMPK phosphorylated at Thr^172^, as i) the molecular weight is the proper to the α subunit of AMPK and ii) this upper band is also recognized with the anti AMPKα antibody used in Figure 1. Negative control was also performed omitting the primary antibody and blot was probed with secondary antibody (anti-rabbit-HRP) only. No bands are detected with the secondary antibody and confirm that bands visualized are due to the anti-phospho-Thr^172^-AMPK antibody used (data not shown). As shown in Figure 2A, a clear band of phospho-Thr^172^-AMPK is detected when spermatozoa are incubated at 38.5°C in TBM or in a medium with calcium and bicarbonate (TCM). The intensity of the AMPK phosphorylated band results dependent on the incubation time at 38.5°C, reaching highest levels of phosphorylation between 30–60 minutes. When the same pools of spermatozoa are incubated either in a TBM or TCM at lower temperature, 17°C (considered as minute 0 in the Figure), which is the routine value for porcine semen preservation with low energy consumption, AMPK phosphorylation is very low or even not detectable. This effect is independent of the incubation time of spermatozoa at 17°C (data not shown). A loading control of protein is showed in lower panel of Figure 2A using an anti-GSK3β antibody, as we have previously shown that level of this protein does not change under these experimental conditions [30].

### The AMPK Inhibitor, Compound C, Effectively Blocks AMPK Phosphorylation in Spermatozoa

A widely used inhibitor of the AMPK activity in somatic cells is the Compound C (CC), a cell-permeable pyrrazolopyrimidine compound that acts as a potent, selective, reversible, and ATP-competitive inhibitor of AMPK. Thus, we initially decided to confirm that CC (30 µM) effectively blocks the phosphorylation (activity state) of AMPK in male germ cells. Based on the range of CC concentrations used in the literature in somatic cells, we initially tried several concentrations of CC (1, 10 and 30 µmol/l) in boar spermatozoa. Our results are showed in Figure 2B and demonstrate not only that AMPK remains phosphorylated at physiological temperature after 24 h of incubation but also that 30 µmol/l CC blocks phosphorylation of this kinase in spermatozoa. Moreover, under these conditions CC does not affect the levels of AMPKα in spermatozoa (data not shown).

### AMPK Inhibition by Compound C Significantly Reduces the Percentage of Motile Spermatozoa

**Figure 3:**
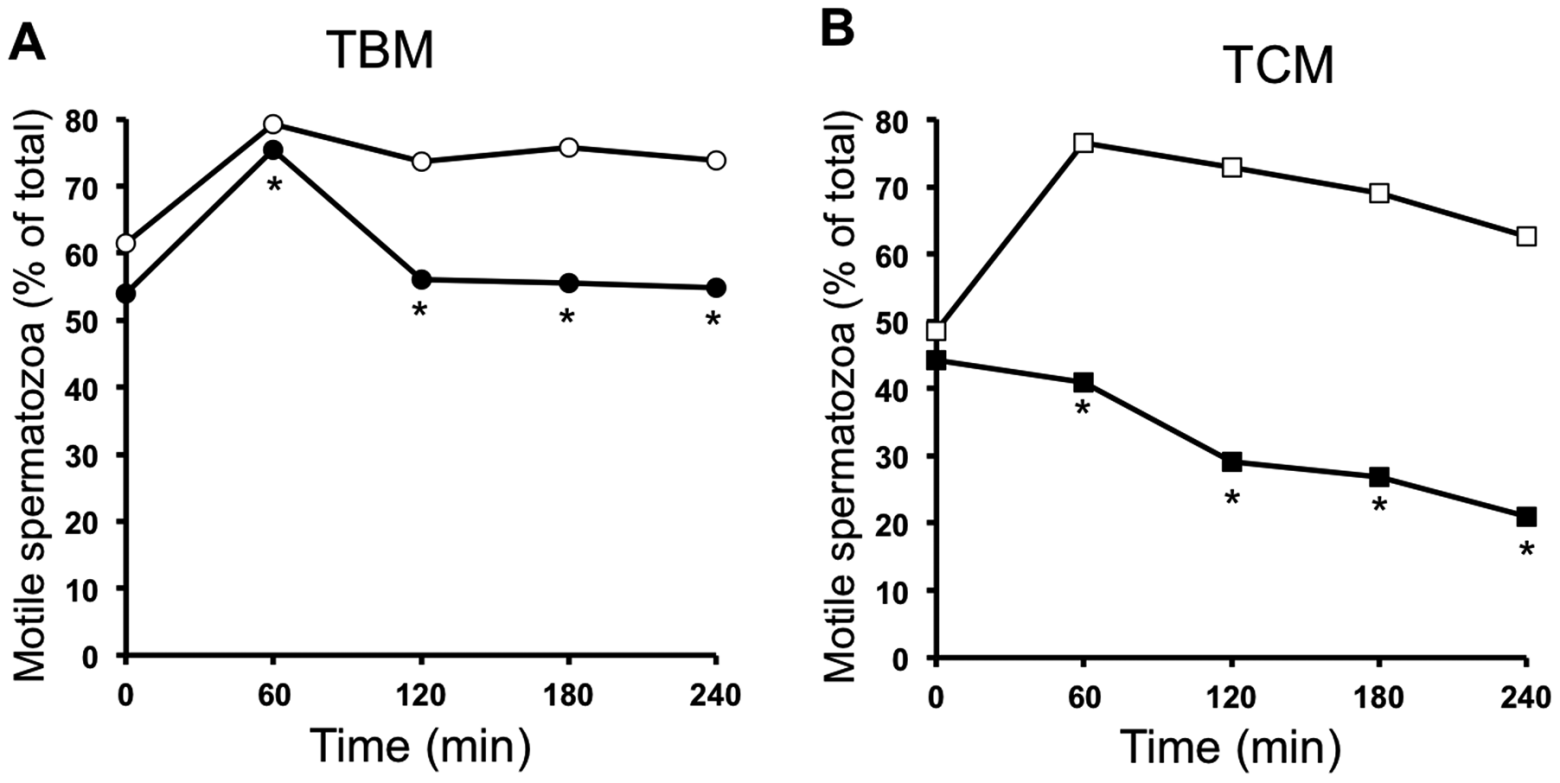
Short time effect (0–4 h) of the AMPK inhibition by CC in the percentage of motile spermatozoa. Spermatozoa were incubated in TBM (A, circles) or TCM medium (B, squares) at 38.5°C in the absence (white) or presence (black) of the AMPK inhibitor, compound C, (CC 30 µM) during 4 h. Samples at 17°C were considered as time 0. The percentage of motile spermatozoa was measured by the ISAS system as described. This experiment was performed at least 6 times and the results express the percentage of motile spermatozoa from the total analyzed (4.000–5.000). Statistical differences were considered when p<0.05 and showed with an asterisk.

To evaluate the effect of the AMPK inhibition by CC (30 µM) in motility, boar spermatozoa were incubated in TBM or TCM, in the presence or absence of CC for different times at physiological temperature 38,5°C. In addition, we have analyzed motility parameters at semen preservation temperature 17°C and included as time 0. It is well described in the literature that the increase in the temperature up to the physiological 38,5°C in boar is a potent stimulator of spermatozoa motility [21]. As observed in Figure 3, short treatment (0–4 h) with CC at 38,5°C leads to a significant reduction in the percentage of motile spermatozoa in either TBM (3A) or TCM (3B). The reduction of the percentage of motile spermatozoa is time-dependent and results statistically significant as rapid as at 1 hour in TCM (3B) or 2 h in TBM (3A). The fall in this percentage caused by AMPK inhibition is clearly greater in TCM (where there is a 66% of reduction in the percentage of motile spermatozoa after AMPK inhibition at 4 h; Fig. 3B) than in TBM (where there is a 26% of reduction in the percentage of motile spermatozoa after AMPK inhibition at 4 h; Fig. 3A).

### AMPK Inhibition Significantly Decreases the Percentage of Rapid Spermatozoa

**Figure 4:**
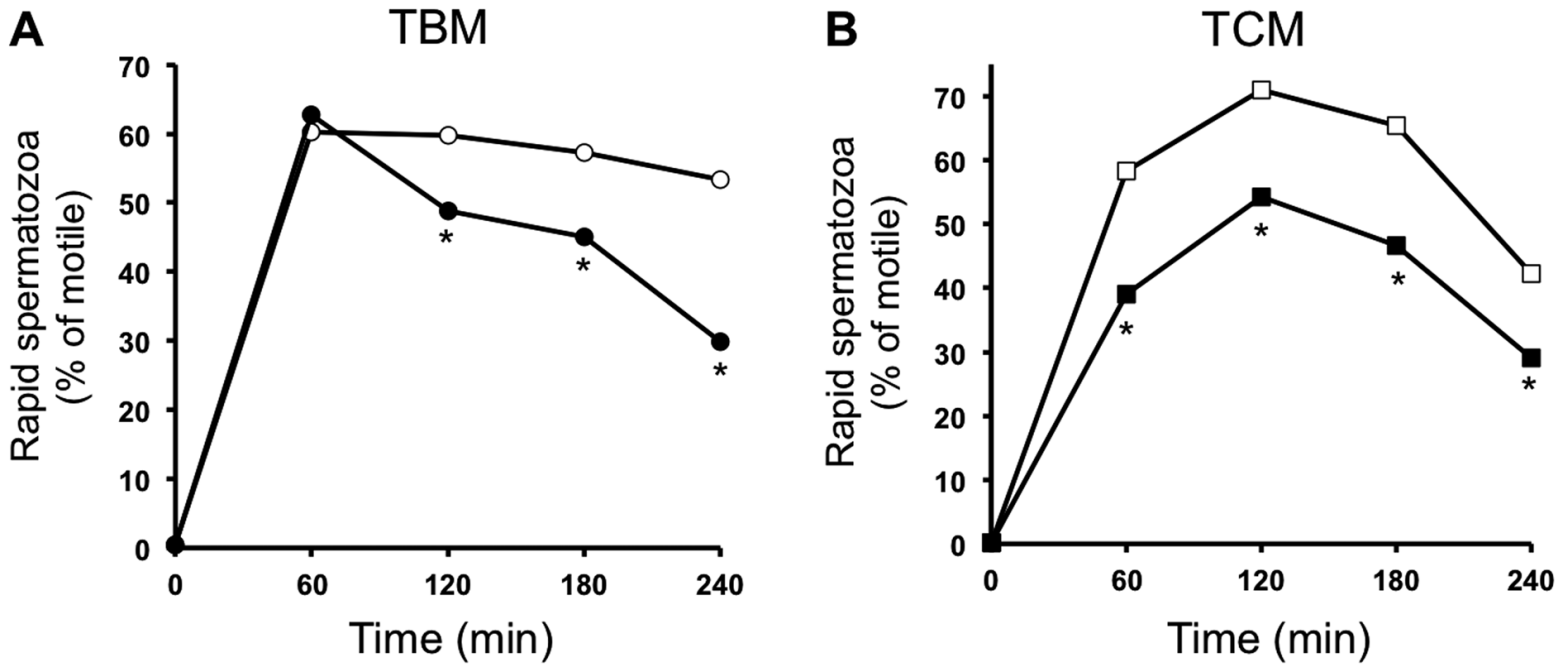
Short time effect (0–4 h) of the AMPK inhibition in the percentage of rapid spermatozoa. Spermatozoa were incubated in TBM (A, circles) or TCM medium (B, squares) at 38.5°C in the absence (white) or presence (black) of the AMPK inhibitor, compound C, (CC 30 µM) during 4 h. Samples at 17°C were considered as time 0. The percentage of those motile spermatozoa with VAP>80 µm/s, defined as rapid spermatozoa, was measured. This experiment was performed at least 6 times and the results express the percentage of rapid spermatozoa from the total spermatozoa motile analyzed (4.000–5.000). Statistical differences were considered when p<0.05 and showed with an asterisk.

Rapid spermatozoa are defined as the percentage of those motile spermatozoa with velocity VAP higher than 80 µm/s. As seen in Figure 4 and according to previous literature [21], the time-course of the percentage of rapid spermatozoa incubated in TBM (Fig. 4A) clearly differs from the time-course in TCM (Fig. 4B). The inhibition of AMPK by CC in either TBM or TCM leads to a significant reduction in the percentage of those motile spermatozoa that move in a rapid manner in a time-dependent manner. However, whereas in TBM the reduction is detected at 2 h and maximum at 4 h of AMPK inhibition (reduction of number of rapid spermatozoa by 44%), in TCM the CC inhibitory effect is detected as rapid as 1 h and remains constant at any time studied (about 30% of reduction of the percentage of rapid spermatozoa).

### AMPK Inhibition Significantly Reduces Spermatozoa Velocities and Affects other Motility Parameters

**Figure 5:**
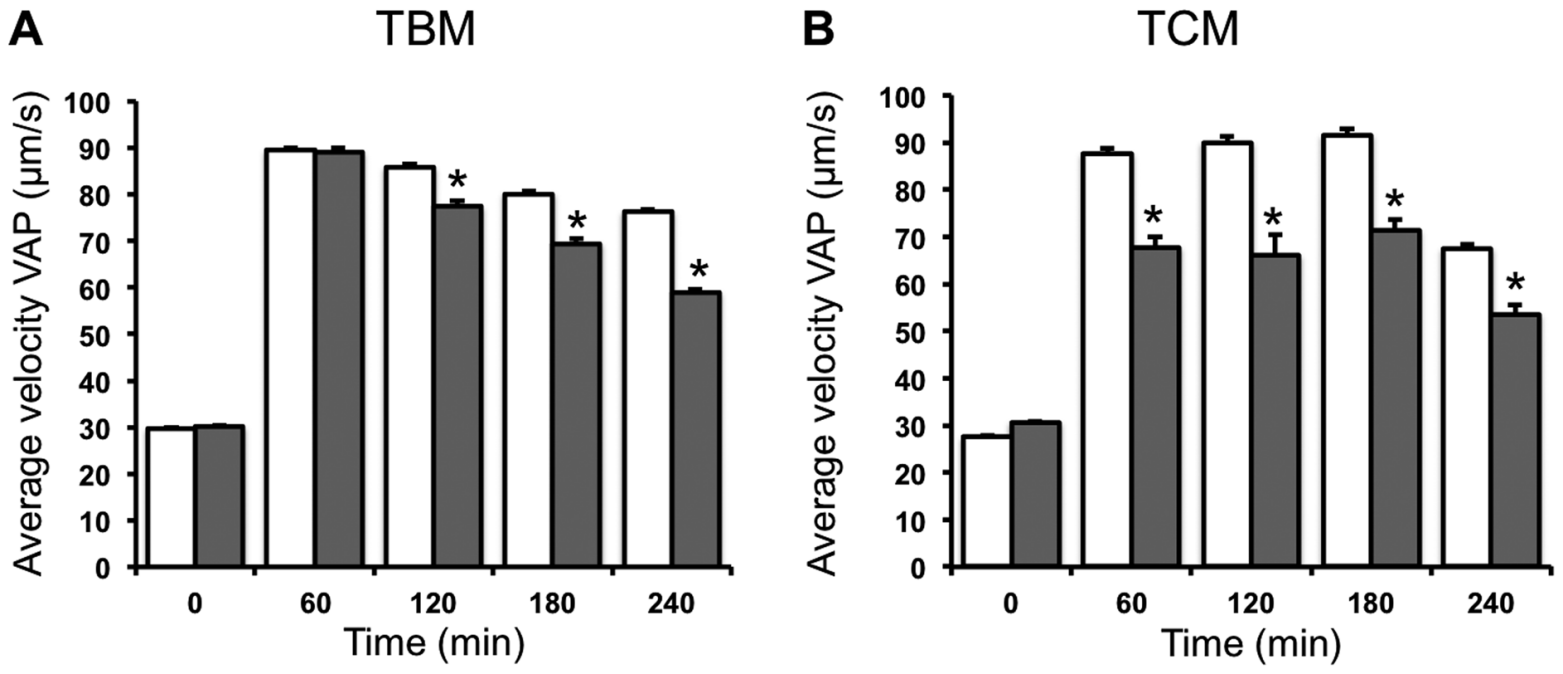
Short time effect (0–4 **h) of the AMPK inhibition in the average path velocity, VAP, of boar spermatozoa.** Spermatozoa were incubated in TBM (A) or TCM medium (B) at 38.5°C in the absence (unfilled bars) or presence (filled bars) of the AMPK inhibitor compound C, (CC 30 µM) during 4 h. Samples at 17°C were considered as time 0. The average path velocity (VAP) was measured and expressed as µm/s. This experiment was performed at least 6 times and the results express the mean ± standard error of the mean. Statistical differences were considered when p<0.05 and showed with an asterisk.

**Figure 6:**
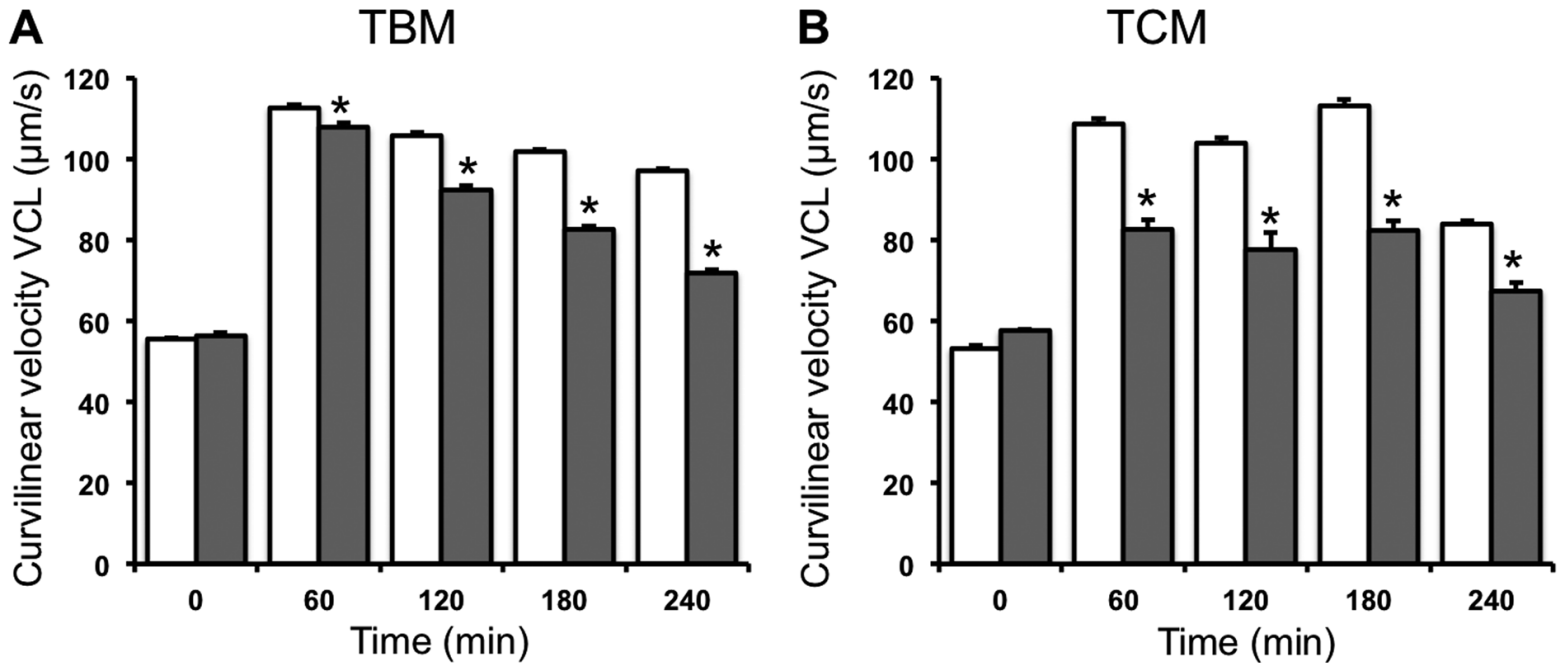
Short time effect (0–4 **h) of the AMPK inhibition by CC in the curvilinear velocity, VCL, of boar spermatozoa.** Spermatozoa were incubated in TBM (A) or TCM medium (B) at 38.5°C in the absence (unfilled bars) or presence (filled bars) of the AMPK inhibitor compound C, (CC 30 µM) during 4 h. Samples at 17°C were considered as time 0. The curvilinear velocity VCL was evaluated and expressed as µm/s. This experiment was performed at least 6 times and results express the mean ± standard error of the mean. Statistical differences were considered when p<0.05 and showed with an asterisk.

As seen in other motility parameters, the time-course of the different velocities analyzed in spermatozoa incubated in TBM clearly differs from the time-course in TCM (Figs. 5 and 6; Tables 1 and 2). Inhibition of AMPK by CC in spermatozoa incubated either TBM or TCM significantly reduces any spermatozoa velocity studied, including average path velocity VAP (Figure 5), curvilinear velocity VCL (Figure 6) and straight linear velocity VSL (Tables 1 and 2) in a time dependent manner. This CC inhibitory effect is detected in TBM (Figs. 5A and 6A; Table 1) at 2 h and the highest reduction in the velocity is achieved at 4 h of AMPK inhibition (VAP by 23%, VCL by 26% and VSL by 13%). However, in TCM (Figs. 5B and 6B; Table 2) the CC inhibitory effect is detected as rapid as 1 h and remains constant at any time studied (22% of reduction of VAP, 24% reduction in the case of VCL and 15% reduction in VSL).

**Table 1:** Short term effect (0–4 h) of the AMPK inhibition by compound C (CC) in boar spermatozoa motility parameters in TBM.

	VSL (µm/s)		LIN (%)		STR (%)		WOB (%)		ALH (µm)		BCF (Hz)	
Min.	TBM	TBM + CC	TBM	TBM + CC	TBM	TBM + CC	TBM	TBM + CC	TBM	TBM + CC	TBM	TBM + CC
**0**	16.9±0.2	17.7±0.3	31.5±0.2	32.6±0.4	56.9±0.3	58.2±0.6	54.4±0.2	54.6±0.3	2.8±0.0	2.8±0.0	6.4±0.1	6.9±0,1
**60**	66.2±0.6	68.3±0.9	54.8±0.4	58.9±0.6*	68.8±0.4	71.6±0.5	75.9±0.2	79.2±0.3*	3.6±0.0	3.4±0.0*	7.2±0.1	7.3±0.1
**120**	67.2±0.7	62.4±1.2*	58.7±0.4	61.6±0.7*	72.4±0.4	73.8±0.7	77.3±0.3	79.3±0.5*	3.3±0.0	2.7±0.0*	7.8±0.1	7.3±0.1*
**180**	62.5±0.5	58.0±1.0*	57.5±0.4	63.6±0.6*	72.4±0.4	76.3±0.6*	75.8±0.2	79.7±0.4*	3.4±0.0	2.6±0.0*	8.1±0.1	7.7±0.1*
**240**	60.6±0.5	49.5±0.8*	58.7±0.4	62.7±0.6*	73.9±0.4	76.7±0.6*	75.9±0.2	78.5±0.4*	3.2±0.0	2.5±0.0*	8.3±0.1	7.9±0.1*

Spermatozoa were incubated in TBM at 38.5°C in the presence or absence of the AMPK inhibitor, CC, (30 µM) during 4 hours. Samples measured at 17°C were considered as time 0. Spermatozoa motility parameters including straight-line velocity (VSL, expressed in µm/s) and coefficients LIN (Linearity coefficient in %), STR (Straightness coefficient in %), WOB (Wobble coefficient in %), ALH (Amplitude of lateral head displacement in µm) and BCF (Beat cross frequency in Hz) were measured by the ISAS system. This experiment was performed at least 6 times and results express the mean ± standard error of the mean. Statistical differences were considered when p<0.05.

**Table 2:** Short term effect (0–4 h) of the AMPK inhibition by compound C (CC) in boar spermatozoa motility parameters in TCM.

	VSL (µm/s)		LIN (%)		STR (%)		WOB (%)		ALH (µm)		BCF (Hz)	
Min.	TCM	TCM + CC	TCM	TCM + CC	TCM	TCM + CC	TCM	TCM + CC	TCM	TCM + CC	TCM	TCM + CC
**0**	14.4±0.3	16.5±0.2	27.8±0.4	29.1±0.3	52.5±0.6	53.2±0.4	52.6±0.3	53.9±0.2	2.8±0.0	2.9±0.0	5.9±0.1	6.2±0.1
**60**	64.0±1.1	54.6±2.1*	53.9±0.6	58.8±1.2*	67.2±0.6	71.4±1.1*	76.9±0.4	78.4±0.8	3.6±0.0	2.7±0.1*	6.8±0.1	5.9±0.2*
**120**	63.9±1.1	73.3±3.1*	57.5±0.6	68.2±1.6*	71.4±0.6	79.3±1.4*	76.5±0.4	83.1±1.0*	3.3±0.0	2.7±0.1*	6.9±0.1	7.3±0.2
**180**	71.8±1.3	62.7±2.2*	61.8±0.7	68.5±1.3*	75.5±0.7	80.4±1.2*	79.4±0.4	82.2±0.8	3.6±0.0	2.5±0.1*	8.2±0.1	7.1±0.2*
**240**	55.1±0.9	45.5±2.1*	60.6±0.3	53.5±1.7	75.1±0.6	74.4±1.6	77.3±0.4	75.9±1.2	2.9±0.0	2.4±0.1*	7.5±0.1	6.7±0.2*

Spermatozoa were incubated in TCM at 38.5°C in the presence or absence of the AMPK inhibitor, CC, (30 µM) during 4 hours. Samples measured at 17°C were considered as time 0. Spermatozoa motility parameters including straight-line velocity (VSL, expressed in µm/s) and coefficients LIN (Linearity coefficient in %), STR (Straightness coefficient in %), WOB (Wobble coefficient in %), ALH (Amplitude of lateral head displacement in µm) and BCF (Beat cross frequency in Hz) were measured by the ISAS system. This experiment was performed at least 6 times and results express the mean ± standard error of the mean. Statistical differences were considered when p<0.05.

Moreover, AMPK inhibition by treatment of spermatozoa with CC incubated in either TBM or TCM for short term (up to 4 h) significantly affects in different ways other spermatozoa motility parameter analyzed: LIN, STR, WOB, ALH and BCF (Tables 1 and 2).

### Effect of the Long-term AMPK Inhibition by CC in Spermatozoa Motility Parameters

As literature describes that effects of AMPK inhibition in somatic cells can be obtained not only a short-term but at long-term [22], we next evaluated the action of AMPK inhibition in spermatozoa motility at longer times than 4 h by treatment with CC for 24 hours (Table 3). Inhibition of AMPK for longer time in TBM induces a higher and significant effect than short term in all spermatozoa motility parameters mentioned. Thus, potent reduction of 82% in the percentage of motile spermatozoa, as well as a full reduction of the percentage of rapid spermatozoa were obtained by long term CC treatment (Table 3). Concomitantly, AMPK inhibition by 24 h causes a clear and significant diminution in any spermatozoa velocity analyzed: VCL as an estimate of instantaneous sperm swimming speed, by 45%, VSL by 61% and VAP by 53%. The rest of spermatozoa motility parameters were also significantly reduced by 24 h of AMPK inhibition with CC.

**Table 3:** Long-term effect (24 h) of the AMPK inhibition by CC in boar spermatozoa motility.

SPERMATOZOA MOTILITYPARAMETERS	TBM	TBM +CC (30 µM)
Motile spermatozoa (%)	58.1^a^	10.1^b^
Rapid spermatozoa (VAP>80 µm/s) (%)	7.7^a^	0.5^b^
VCL (µm/s)	56.2±0.4^a^	31.2±0.9^b^
VSL (µm/s)	38.1±0.4^a^	14.9±0.6^b^
VAP (µm/s)	45.6±0.4^a^	21.4±0.6^b^
LIN (%)	64.2±0.5^a^	49.5±1.3^b^
STR (%)	78.5±0.4^a^	65.8±1.4^b^
WOB (%)	78.9±0.3^a^	70.9±0.9^b^
ALH (µm)	2.0±0.01^a^	1.7±0.03^a^
BCF (Hz)	8.1±0.07^a^	4.1±0.1^b^

Spermatozoa were incubated in TBM at 38.5°C in the presence or absence of the AMPK inhibitor, CC, (30 µM) during 24 h. Spermatozoa motility parameters including the percentages of motile and rapid spermatozoa, curvilinear velocity (VCL), average path velocity (VAP), straight-line velocity (VSL) and coefficients LIN, STR, WOB, ALH and BCF were measured by the ISAS system. This experiment was performed at least 5 times and results express the mean ± standard error of the mean. Statistical differences are shown as a, b when p<0.0001 between treatments (columns).

### Effect of the AMPK Inhibition by CC in the Viability of Boar Spermatozoa

We have studied the effect of AMPK inhibition by CC in spermatozoa viability in order to correlate it with motility studies and in addition to know whether CC treatment might cause spermatozoa side effects that lead to germ cell death. According to our previous results [21], viability of boar spermatozoa is sensitive to both temperature and the presence of Ca^2+^ and/or bicarbonate in the medium in a time dependent manner, as confirmed in Table 4. Thus, spermatozoa viability measured as the number of SYBR-14^+^/IP^−^ spermatozoa, decreases after 4 hours at 38,5°C by 30% in TBM and by 42% in TCM. Short-term exposure to compound C does not affect in a significant manner the percentage of viable spermatozoa in any experimental condition (Table 4). However, a slight but reproducible effect of CC preventing the loss of cell viability is observed in TBM, were 66% of sperm cells remain viable after 4 h with CC versus 59% in the absence of CC. This protective effect of compound C in the loss of spermatozoa viability in TBM becomes significant at 24 h of treatment (Table 4), where 63% of spermatozoa remain viable in the presence of compound C compared with 49% in its absence.

**Table 4:** Effect of the AMPK inhibition by compound C in spermatozoa viability.

Cell Viability	TBM	TBM +CC (30 µM)	TCM	TCM +CC (30 µM)
17°C	83.22±2.0^a^	83.22±2,.^a^	82.57±1.9^a^	82.57±1.9^a^
1 h (38.5°C)	77.22±1.4^a^	77.80±2.6^a^	70.72±2.1^a^	67.30±3.1^a^
4 h (38.5°C)	58.97±6.5^a^	66.32±3.2^a^	48.20±5.7^a^	51.25±5.7^a^
24 h (38.5°C)	48.77±2.1^a^	62.49±1.9^b^	54.83±3.7^a^	56.63±3.4^a^

Spermatozoa were incubated in TBM or TCM medium at 17°C or 38.5°C in absence or presence of the AMPK inhibitor CC (30 µM) for different times (1–24 h). Spermatozoa viability was measured by flow cytometry using SYBR-14 and IP as probes. This experiment was performed at least 4 times and the results expressed as percentage of viable cells are shown as mean ± standard error of the mean. Statistical differences are shown as a,b when p<0.001 between treatments (columns).

## Discussion

The control of cell metabolism in spermatozoa is achieved by dynamic mechanisms able to adapt to environmental changes and related with cellular structures such as mitochondria or plasmalemma [23]. Therefore, regulation of the energy levels during changing extracellular conditions, such as those leading to oocyte fertilization, is of essential importance in the understanding of spermatozoa function. In mammalian tissues the protein AMPK controls metabolism [8], [24] by activating metabolic pathways that produce ATP and simultaneously by inhibiting those pathways that consume ATP [9], [10], however, to date there are not data about the presence or function of AMPK in male germ cells.

The present study shows for the first time that the metabolic sensor AMPK is expressed in spermatozoa. The AMPK antibody against α subunit recognizes two bands with close molecular weight in boar spermatozoa, in agreement with the data obtained in vascular smooth muscle from the same specie [25]. As the AMPKα antibody reacts with two α isoforms of human origin (α1 and α2), these two reactive bands in porcine spermatozoa likely include α1 and α2 isoforms of AMPK. It is interesting to mention that the expression level of AMPKα protein detected in male germ cells is likely higher than in somatic cells derived from porcine heart, brain and lung, where a unique reactive band is detected.

Our results demonstrate that AMPK is phosphorylated at Thr^172^, and therefore subsequently activated, at physiological temperature of boar spermatozoa (38,5°C). Although the phosphorylation level of the kinase varies with the time of incubation at 38,5°C increasing mainly in the first 60 minutes, AMPK phosphorylation (activation) remains clearly detectable after 24 h of treatment. Our results suggest that phosphorylation of AMPK in spermatozoa does not require Ca^2+^ and/or bicarbonate in the extracellular medium to occur, as it is detected either in presence or in absence of these ions. The fact that AMPK is phosphorylated at physiological temperature and that phosphorylation occurs both in TBM and TCM, which contains Ca^2+^ and bicarbonate, suggests that activation of this kinase likely occurs *in vivo* under those physiological conditions of spermatozoa when transiting through the female reproductive tract that are experimentally mimicked by these two media.

Potential side effects of the treatment with a pharmacological inhibitor as CC can be rule out in this study as spermatozoa viability was analyzed in parallel experiments. In this sense, even the longest time of CC incubation (24 h) does not cause any side effect that might lead to a loss in spermatozoa viability. By contrary, our results suggest that AMPK activity might be involved in the modulation of the control of spermatozoa viability under some conditions, as compound C significantly prevents the loss in viability induced by time and temperature in TBM. Interestingly, compound C has not effect in the viability of spermatozoa incubated in TCM, which suggest that the possible regulatory role of AMPK in germ cell viability is dependent on the presence of Ca^2+^ and/or bicarbonate in the medium. This is not surprising, as Ca^2+^ is a major regulator of AMPK activity in somatic cells.

The fact that CC treatment causes a clear and significant reduction of the percentage of motile spermatozoa implies that AMPK inhibitor induces a potent increase in the number of motionless spermatozoa. Under these conditions, those remnant spermatozoa that still are motile move with significantly lower speed, as AMPK inhibition leads to a significant decrease in the number of rapid spermatozoa (VAP>80 µm/s). Concomitantly, AMPK inhibitor reduces spermatozoa curvilinear velocity VCL (an estimate of instantaneous sperm swimming speed) and average path velocity VAP. Although we rule out potential side effects of CC in spermatozoa, we cannot exclude the possibility that the effects observed on spermatozoa motility following CC treatment might result from disruption of other pathways, independently of AMPK. Therefore, our results, showing that the AMPK inhibitor exerts a potent anti-motility effect in boar spermatozoa, evaluated by different parameters, allow us to suggest that AMPK activity is likely necessary for optimal spermatozoa motility. Moreover, they also suggest that AMPK is not sufficient to achieve proper sperm motility. This idea is expected as our previous studies and others have established that the control of spermatozoa motility is achieved by the contribution, in a convergent or parallel manner, of several signalling kinase pathways [26], [27], [28], [29], [30].

The cellular action most related to spermatozoa motility induced by the AMPK inhibitor CC found in the literature is the inhibition of cell migration in different type of cancer cells such as glioma [31] or ovarian cancer cells [32]. As mentioned, there are not previous reports about the role of AMPK in spermatozoa motility.

Having in mind the energy-regulating role of AMPK in somatic cells, it logical to assume that it may play a role in those spermatozoa functions that are particularly dependent on the energy levels, such as motility. Specifically, AMPK plays a central role in the maintenance of cell energy levels by regulating among others pathways, the glycolysis [33]. Thus, as spermatozoa motility is totally dependent on ATP supply, generated mainly via glycolylis [34] or by mitochondrial activity, it is reasonable to assume that AMPK activity is likely required for a optimal spermatozoa motility. In this regard, it is interesting to mention that intracellular mediator Ca^2+^, which plays an essential role in spermatozoa motility [35], exerts a modulator function in the AMPK- regulated spermatozoa motility. However, we cannot exclude a possible non-metabolic effect of the AMPK pathway that could control spermatozoa motility in a parallel or synergistic way, as AMPK is a serine/threonine kinase with several known downstream substrates and therefore may regulate processes outside of cell metabolism. Recently, it has been demonstrated that the kinase TSSK2, which belongs to the AMPK branch in the human kinome tree and is expressed in spermatozoa [17], phosphorylates *in vitro* the axoneme central apparatus protein called SPAG16L [36], that is essential for flagellar motility in mouse spermatozoa [37]. Thus, it can be conceivable that its close related protein, AMPK might phosphorylate downstream substrates involved in the axoneme central apparatus similar to SPAG16L, or in other related structures that are essential for spermatozoa flagellar motility.

Our study points to a regulatory role of AMPK for proper spermatozoa motility, and therefore it is important to understand what factor(s) trigger the activation of AMPK and how this activation is modulated in male germ cells. We believe that this study opens future investigations about AMPK in spermatozoa and we consider that further work is necessary to elucidate mentioned key questions. In conclusion, the present study demonstrates that AMPK protein is expressed in boar spermatozoa and is phosphorylated at Thr^172^ (active) under physiological conditions of these male germ cells. As inhibition of AMPK clearly causes a potent inhibition of spermatozoa motility, our findings suggest that AMPK activity likely plays an important role in the regulation of optimal spermatozoa motility. Motility is essential for the ultimate function of spermatozoa, oocyte fertilization, therefore we propose for the first time that AMPK protein might play an important and necessary regulatory role in the mammalian spermatozoa function.

## Materials and Methods

### Chemicals and Sources

Complete, EDTA-free, protease inhibitor cocktail was purchased from Roche Diagnostics (Penzberg, Germany). Tris/Glycine/SDS buffer (10X) and Tris/Glycine buffer (10X) from Bio-Rad (Richmond, CA). Hyperfilm ECL was from Amersham (Arlington Heights, IL). Enhanced chemiluminescence detection reagents, anti-mouse IgG-horseradish peroxidase conjugated and anti-rabbit IgG-horseradish peroxidase conjugated were from Pierce (Rockford, IL). Nitrocellulose membranes were from Schleicher & Schuell, BioScience (Keene, NH). Compound C (6-[4-(2-Piperidin-1-ylethoxy) phenyl]-3-pyridin-4-ylpyrazolo[1,5-a]pyrimidine) were from Sigma-Aldrich® (St Louis, MI, USA). Anti-AMPKα and anti-GSK3β antibodies were from Cell Signaling (Beverly, CA). Anti-P-Thr^172^-AMPK antibody was from Santa Cruz Biotechnology (Santa Cruz, CA, USA). Live/dead spermatozoa viability kit from Molecular Probes (Leiden, The Netherlands); Coulter Isoton II Diluent from Beckman coulter Inc. (Brea, CA, USA).

### Spermatozoa Incubation Media

Tyrode’s basal medium (TBM) was prepared as following: 96 mmol/l NaCl, 4.7 mmol/l KCl, 0.4 mmol/l MgSO_4_, 0.3 mmol/l NaH_2_PO_4_, 5.5 mmol/l glucose, 1 mmol/l sodium pyruvate, 21.6 mmol/l sodium lactate, 20 mmol/l HEPES (pH 7.45) and 3 mg/ml BSA. A variant of TBM medium, which includes direct activators of spermatozoa adenylyl cyclase sAC, was made by adding 1 mmol/l CaCl_2_ and 10 mmol/l NaHCO_3_ and equilibrated with 95% O_2_ and 5% CO_2_ and termed Tyrode’s complete medium (TCM). All Tyrode’s mediums were made on the day of use and maintained at pH 7.45 with an osmolarity of 290–310 mOsm kg^−1^.

### Collection and Washing of Semen

Semen from Duroc boars (2–4 years old) was used. Animals were housed at a commercial insemination station (Tecnogenext, S.L, Mérida, Spain) and maintained according to institutional and European regulations. All boars were housed in individual pens in an environmentally controlled building (15–25°C) and received the same diet. Artificial insemination using preserved liquid semen from boars demonstrated their fertility. Fresh ejaculates were collected with the gloved hand technique and stored at 17°C before use and, in order to minimize individual boar variations, samples from up to 3 animals were pooled using semen from no less than 12 boars in different combinations. Only semen pools with at least 80% morphologically normal spermatozoa were used. Semen was centrifuged at 2000 *g* for 4 minutes, washed with PBS and placed in TBM or TCM medium. Samples of 1.5 ml containing 120×10^6^ spermatozoa/ml were incubated at 38,5°C in a CO_2_ incubator for different times for western blotting analysis and lower volume (0.5 ml) was used in samples prepared for evaluation of motility. When required, a preincubation of spermatozoa with compound C was performed for 1 hour at RT. In order to minimize possible experimental variations, every condition/treatment studied was performed in the same semen pool. When necessary, a control with the final concentration of the solvent (DMSO 0.1%) was included.

### Western Blotting

Spermatozoa under different treatments were centrifuged 20 s at 7000 *g*, washed with phosphate buffered saline (PBS) supplemented with 0.2 mM Na_3_VO_4_ and then lysated in a lysis buffer consisting in 50 mmol/l Tris/HCl, pH 7.5, 150 mmol/l NaCl, 1% Triton X-100, 1% deoxycholate, 1 mmol/l EGTA, 0.4 mmol/l EDTA, protease inhibitors cocktail (Complete, EDTA-free), 0.2 mmol/l Na_3_VO_4_, and 1 mmol/l PMSF by sonication for 5 s at 4°C. After 20 minutes at 4°C samples were centrifuged at 10.000 *g* (15 minutes, 4°C) and the supernatant (lysate) was used for analysis of protein concentration. Proteins from porcine spermatozoa lysates were resolved by SDS-PAGE and electro-transferred to nitrocellulose membranes. Western blotting was performed as previously described [38] using anti AMPKα (1∶1.000), anti phospho-Thr^172^-AMPKα (1∶500), anti GSK3β (1∶2.000) polyclonal antibodies as primary antibodies.

### Evaluation of Spermatozoa Motility by Computer Assisted Sperm Analysis (CASA) System

After incubation of spermatozoa in TBM or TCM with 5% CO_2_ at 38,5°C during different times, a total of 2 µl of sample was placed in a pre-warmed counting chamber (Leja®, Luzernestraat, The Netherlands). Sperm motility analysis is based on the examination of 25 consecutive digitalized images obtained from a single field using a X10 negative-phase contrast objective, and at least 400 spermatozoa per sample were analyzed. Images were taken with a time lapse of 1 s and objects incorrectly identified as spermatozoa were eliminated from the analysis. Motility parameters evaluated with the ISAS® program (Projectes i Serveis R+D, SL; Valencia, Spain) were as following: VCL (curvilinear velocity, in µm/s), VSL (straight-line velocity in µm/s), VAP (average path velocity, in µm/s), LIN (linearity coefficient in %), STR (straightness coefficient in %), ALH (amplitude of lateral head displacement in µm), WOB (wobble coefficient in %), BCF (Beat cross frequency in Hz). Those spermatozoa with VAP<10 µm/s were considered immobile, while spermatozoa with a velocity >10 µm/s were considered mobile; spermatozoa with a VAP velocity >80 µm/s were considered as rapid spermatozoa. Spermatozoa motility was considered progressive (MP) when STR >80%.

### Analysis of Spermatozoa Viability by Flow Cytometry

As described previously [21], fluorescent staining using the Live/Dead Sperm Viability kit was performed to assess porcine spermatozoa viability. Briefly, 5 µl of SYBR-14 (2 µM) and 10 µl of PI (5 µM) were added to 500 µl of spermatozoa sample diluted to 35×10^6^ cells/ml in isotonic buffered diluent and incubated 20 min at room temperature in the darkness. After incubation, sperm cells were analyzed in the flow cytometer and results were expressed as the average of the percentage of SYBR14-positive and propidium iodide-negative spermatozoa ± SEM. Flow cytometry analysis was performed using a Coulter EPICS XL-MCL flow cytometer (Beckman Coulter Ltd.) The fluorophores were excited by a 200 mV argon ion laser operating at 488 nm. A total of 10.000 gated events (based on the forward scatter and side scatter of the sperm population recorded in the linear mode) were collected per sample with sample running rates of approximately 500 events/s. Fluorescence data were collected in the logarithmic mode. The fluorescence values of SYBR-14 were collected in the FL1 sensor using a 525 nm bad pass (BP) filter. Propidium Iodide (PI) fluorescence was collected in the FL3 sensor using a 620 nm BP filter. Flow cytometry data were analysed using a FacStation computer and EXPO™ 32 ADC software (Beckman Coulter, Inc.).

### Statistical Analysis

The mean and standard error of the mean were calculated for descriptive statistics, whenever was possible. The effect of treatment and incubation time on the motility variables was assessed using a General Linear Model. To analyze the percentage of motile, progressive and rapid spermatozoa from the total of spermatozoa analyzed we used the Pearson Chi-square test. All analyses were performed using SPSS v11.0 for MacOs X software (SPSS Inc. Chicago, IL). The level of significance was set at p<0.05.
